# A Virtual Tai Chi Intervention for Older Adults with Mobility Disabilities: Results from a Single-Arm Clinical Trial with the TechSAge Tele Tai Chi Program

**DOI:** 10.3390/healthcare14121756

**Published:** 2026-06-18

**Authors:** Tracy L. Mitzner, Elena T. Remillard, Kara T. Mumma, Michael W. Boyce

**Affiliations:** 1 Person in Design, Atlanta, GA 30306, USA; 2 Center for Inclusive Design and Innovation, Georgia Institute of Technology, Atlanta, GA 30318, USA; elena.remillard@design.gatech.edu (E.T.R.); kara.mumma@design.gatech.edu (K.T.M.); 3 Boyce Human Performance, LLC, Shelton, CT 06484, USA; michael@boycehumanperformance.com

**Keywords:** aging, mobility disability, physical activity, social connectedness, tai chi, clinical trial

## Abstract

**Highlights:**

**What are the main findings?**

- An 8-week evidence-based in-person Tai Chi program for older adults, *Tai Chi for Arthritis and Fall Prevention*, was successfully translated to a virtual intervention with a social component for people aging with mobility disabilities.
- The virtual translation was feasible, rated as acceptable and enjoyable by participants, and was associated with increases in physical activity and social participation.

**What are the implications of the main findings?**

- The *TechSAge Tele Tai Chi program* has the potential to reduce barriers to physical activity and social participation for people who are aging with mobility disabilities.
- The results support the feasibility of translating in-person health and wellness interventions to virtual platforms to increase the reach of those programs, particularly to those who have significant barriers to in-person attendance.

**Abstract:**

**Background/Objectives**: Telewellness programs can expand access to exercise and social opportunities for older adults, especially those with mobility disabilities. The *TechSAge Tele Tai Chi* clinical trial assessed whether the *Tai Chi for Arthritis and Fall Prevention* program was feasible and acceptable when delivered in a virtual format for adults aging with mobility disabilities, and examined pre-to-post changes in two primary outcomes: physical activity and social connectedness. **Methods**: The *TechSAge Tele Tai Chi* study was a single-arm clinical trial. Sixty community-dwelling adults (60–77 years of age; *M* = 69.2, *SD* = 4.8) with self-identified long-term mobility disability (≥10 years) joined the virtual classes from home twice a week for 8 weeks. Participants exercised along with pre-recorded video lessons and engaged in guided social discussion. Assessments at baseline, post-intervention, and 1-month follow-up were analyzed with linear mixed models. **Results**: Leisure physical activity (PASIPD) increased significantly, with back-transformed marginal means rising from 14.2 MET h/wk at baseline to 28.7 MET h/wk post-intervention (*p* < 0.001). The Social Participation subscale of social connectedness also increased from baseline to post-intervention (*p* = 0.014); the overall social-connectedness composite did not change significantly. The virtual translation was feasible with high intervention fidelity and adherence, and participants reported high acceptability, satisfaction, enjoyment, and intention to continue. **Conclusions**: Adults aging with mobility disabilities can safely and successfully participate in virtual group tai chi with appropriate modifications and technology support.

## 1. Introduction

There has been tremendous growth in the popularity of telewellness programs, which use technology to deliver physical and social wellness programs virtually. The flexible format enables participation from home or in other preferred locations (e.g., community buildings) and expands access to specialized content (e.g., adapted fitness programs). Telewellness programs offer a promising approach to supporting health and quality of life among older adults, which can ultimately support aging in place. Videoconference-delivered exercise instruction has demonstrated feasibility, safety, and effectiveness in improving physical function, muscle strength, emotional status, and quality of life in adults over 65, as compared to no intervention [1].

Telewellness could be especially impactful for people who are aging with mobility disabilities. An estimated 20% of older Americans (ages 65 and over) report having a mobility disability [2]. This equates to roughly one in every five older adults experiencing serious challenges with walking or climbing stairs. Furthermore, the risk of mobility impairment increases with age [3]. Mobility disability puts people at risk for being sedentary [4,5], with fewer than 40% of US adults with mobility impairment meeting physical activity guidelines [6]. Aging is associated with an increased risk of social isolation [7,8]. One study found that one in three adults aged 50–80 reported feeling isolated from others (29% some of the time, 5% often) in the past year [9]. Given that disability is also associated with social isolation [10], people aging with mobility disabilities may be at even greater risk, though direct estimates for this specific subgroup are needed to confirm this.

These deficits are driven in part by barriers experienced by older adults with long-term mobility disabilities, which can negatively impact their ability to participate in activities independently and age in place [11,12]. With respect to physical activities, people aging with mobility disabilities encounter challenges such as a lack of specialized instruction and adaptability [11], as well as transportation challenges and inaccessible facilities [13,14,15]. This population also experiences barriers to engaging in social activities due to factors such as lack of transportation and assistance from others, limited financial resources, inaccessible spaces, and physical challenges (e.g., joint/muscle pain) [16].

Physical activity and social engagement are both significant contributors to successful aging (e.g., self-rated health, subjective well-being [17]). Exercise is associated with less pain, fatigue, and depression, as well as greater self-efficacy in managing health and social participation for people aging with mobility disability [18]. Social engagement is associated with positive psychological functioning [19]. Therefore, interventions to support physical and social wellness are critical for enabling adults aging with mobility disabilities to age successfully. Physical activity interventions have been shown to reduce major mobility disability among older adults at risk for disability [20] and improve mobility and physical functioning in older adults with mobility problems, disabilities, and/or multi-morbidities [21]. Targeted social interventions have shown decreased loneliness, reduced depression, increased social activity frequency, and reduced barriers to socializing [22,23].

Whereas regular physical exercise is effective in improving functional health outcomes, group-based exercise can offer added benefits such as reduced social isolation [24,25,26], greater group cohesion, higher satisfaction and self-perceived health, and stronger adherence as compared to exercising alone [27,28,29]. For older adults with physical limitations specifically, social engagement during physical activity may reinforce attendance and motivation while reducing future functional loss [30]. Similarly, supervised exercises (e.g., community-based in-person or remote classes with instructors) are more effective than non-supervised exercises (e.g., home-based with printed or video instruction) [31,32,33,34].

One promising type of group exercise to support the physical and social wellness of older adults with mobility disabilities is tai chi. Tai chi is a mind–body exercise that has been shown to be effective in improving physical, psychosocial, and cognitive outcomes. Physical improvements include reducing the number of falls [35,36,37] and improving balance [38], gait [38,39,40], flexibility [38], mobility, lower limb strength, knee extension strength [41], and sleep [42]. Psychosocial improvements include reducing fear of falling [37,40], fatigue and depression [38,40], and quality of life [38]. Improvements in cognitive function have been found for orientation, registration, attention, calculation, recall, language, and overall neuropsychological performance [43,44,45]. Studies have shown the benefits of tai chi for a range of specific populations of older adults, including those with frailty [37], sarcopenia [37], chronic stroke [46], insomnia [42], central obesity [47], hypertension [48], multiple sclerosis [38], knee osteoarthritis [49], mild cognitive impairment [43,50,51], diabetes mellitus [52], and Parkinson’s disease [53]. For a systematic review and meta-analysis of randomized controlled trials, see [54].

Most community-based physical activity interventions are delivered in person and are not designed for people with mobility disabilities. Given the synergistic effects of group-based physical activity, there is an opportunity to support this population with an evidence-based, accessible, virtual, and social physical activity intervention. While general evidence supports tai chi for older adults, programs tailored to people aging with mobility disabilities are scarce, and few have been adapted for virtual delivery. *Tai Chi for Arthritis and Fall Prevention (TCAFP)* is a low-impact, evidence-based program through the Tai Chi for Health Institute (https://taichiforhealthinstitute.org) that is especially well-suited for people aging with mobility disabilities. The robustness of TCAFP is supported by multiple randomized controlled trials demonstrating its efficacy for fall prevention and improvements for balance, pain, and physical function [55,56]. *TCAFP* is a Title III-D Highest Tier Evidence-based Health Promotion/Disease Prevention Program recommended by the Administration on Community Living and the Centers for Disease Control and Prevention. The program has been adopted by a wide range of Area Agencies of Aging across the United States and is growing in popularity globally. The seated version of this program was designed to accommodate older adults with a range of disabilities and health conditions.

The objective of the *TechSAge Tele Tai Chi* clinical trial was to assess the feasibility and acceptability of TCAFP (seated version) when implemented as a virtual intervention with a social component for adults aging with mobility disabilities. We also examined pre-to-post changes in two primary outcomes: physical activity and social connectedness. To our knowledge, no other clinical trial has tested an intervention combining physical activity and social engagement specifically for older adults aging with mobility disabilities. We also examined secondary outcomes of exercise self-efficacy, falls efficacy, quality of life, depression, and pain, as well as other outcomes (i.e., technology acceptance and class satisfaction). We hypothesized that participants would demonstrate significant pre–post improvements in physical activity and social connectedness as primary outcomes. Given the intervention structure, we expected the social participation subscale to be especially sensitive to change, while acknowledging that constructs such as loneliness and network size may require longer or more intensive social interventions to show significant improvement.

## 2. Materials and Methods

The *TechSAge Tele Tai Chi* study was a single-arm clinical trial conducted through the Georgia Institute of Technology. The primary aims were to assess the feasibility and acceptability of a novel virtual adaptation; as such, we chose a single-arm design, which requires shorter duration and a smaller sample size, which can save costs, in comparison to a larger, randomized controlled trial [57]. In the absence of prior evidence on virtual tai chi specifically adapted for adults aging with long-term mobility disabilities, a single-arm feasibility trial was the appropriate first step. Consistent with the single-arm design, blinding of participants and assessors was not implemented. Participants were aware they were receiving the intervention, and outcome assessments were conducted by trained study staff who were not independent of the research team.

All intervention sessions and assessments were delivered remotely, with participants joining from their own homes using a computer or tablet. Assessments were conducted at three timepoints: pre-intervention, post-intervention, and 1-month follow-up. The study was approved by the Georgia Institute of Technology’s Institutional Review Board (H19560; approved 9 March 2020), and all participants provided informed consent. The study was registered on ClinicalTrials.gov (ID NCT04696887; see Supplementary Materials).

### 2.1. Participants and Recruitment

The sample was a convenience sample and included 60 individuals between the ages of 60–80 who self-identified as having a mobility disability for at least 10 years (i.e., use a mobility aid or have serious difficulty walking or climbing stairs); passed the Physical Activity Readiness Questionnaire (PAR-Q [58]) or provided a letter from a healthcare provider stating that they are approved to participate in the seated exercise program; had access to a computer or tablet with a webcam; had internet access for the next six months; had basic computer skills (e.g., comfortable checking email, watching videos online); lived in the United States; and was conversational in English. Participants were ineligible if they were blind or deaf; were unavailable for the duration of the study; practiced tai chi during the past 6 months; or did not pass the Telephone Interview for Cognitive Status (TICS [59]), by scoring lower than 26. Note that the technology-related inclusion criteria (e.g., computer/tablet access, internet access, and basic computer skills) likely resulted in some self-selection bias, enrolling a sample that was more technologically experienced and motivated than the broader population of older adults aging with mobility disabilities. Recruitment methods included word-of-mouth, participant registries (i.e., TechSAge), social media, community flyers, and outreach to local and national organizations for people with mobility disabilities or with specific related conditions (e.g., multiple sclerosis, post-polio syndrome). The consort diagram and a detailed description of the intervention development and study methods are presented in an earlier publication [60].

### 2.2. Procedure

Eligible participants completed an individual orientation with a researcher on Zoom. During the orientation, the researcher provided basic training on Zoom and completed an environmental checklist to assess any potential safety concerns and suggest any audio or video adjustments. If a participant experienced technical issues during the orientation (e.g., poor Wi-Fi), the researcher helped to troubleshoot a solution. If the problem could not be resolved after significant troubleshooting, the individual was deemed ineligible. Eligible participants provided informed consent and were placed into class cohorts varying in size from 6 to 9 participants. There were 8 total cohorts that completed the intervention over a 2.5-year period. Although each cohort was a convenience sample, there was an informal effort to include a mix of gender and racial representation when creating the class cohorts (e.g., a goal of at least two male participants per class). This process was not systematic or randomized, and gender and racial composition still varied across cohorts.

Participants received up to $60 total in compensation: $20 for completing the pre-intervention assessment, $30 for completing the post-intervention assessment and interview, and $10 for completing the follow-up questionnaire.

### 2.3. Intervention Components

*TCAFP* was developed by the Tai Chi for Health Institute. It is a low-impact mind–body exercise program that involves gentle movements and controlled breathing. The program was designed for people with and without arthritis, as well as those at risk of falling. It offers seated modifications to accommodate people with a range of abilities, including those who are unable to stand. The *TechSAge Tele Tai Chi* intervention utilized the seated version of the program as it was appropriate for our target population of people aging with long-term mobility disabilities.

Because we were adapting the *TCAFP* program to a new and specific population, we established an adaptation committee to guide us in any necessary modifications. The committee included members of the target population, a representative from the Tai Chi for Health Institute, a researcher with expertise in exercise among people aging with mobility disabilities (PhD, MPT, ATP), and a researcher with expertise in technology design for older adults (PhD). Guided by the adaptation committee and in line with instructions from the Tai Chi for Health Institute, we added modifications for participants who were not physically able to participate in a movement by instructing them to visualize the movement. This guidance was rooted in the literature showing that motor imagery activates neurons similar to those activated by physical movements [61] and that motor imagery and execution training have a comparable training curve [62].

We delivered the intervention to cohorts of 6 to 9 participants who joined the class using Zoom. Classes met twice a week for 8 weeks. Participants were provided with an instruction manual as well as training on using Zoom during their orientation. Class facilitators, who were certified to teach the *TCAFP* program, moderated the discussion and provided supplemental support (e.g., suggested movement modifications). The technical moderator was responsible for broadcasting the pre-recorded exercise lessons on Zoom, monitoring participant attendance, completing a fidelity checklist, and addressing technical issues that occurred during the session.

**Figure 1:**
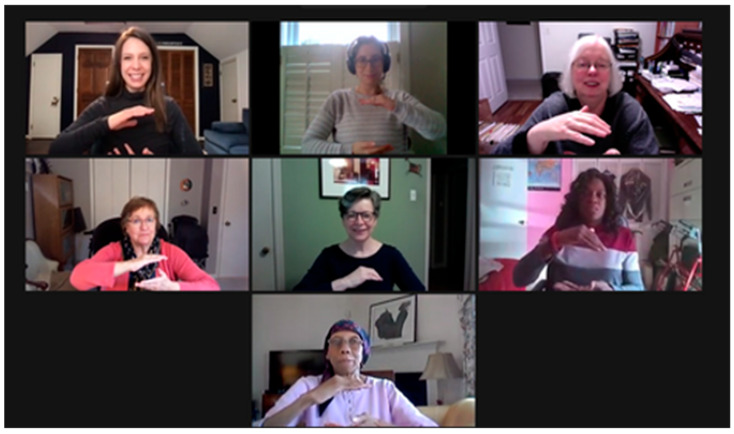
Class facilitator (upper left), technical moderator (upper middle), and participants during social time.

**Figure 2:**
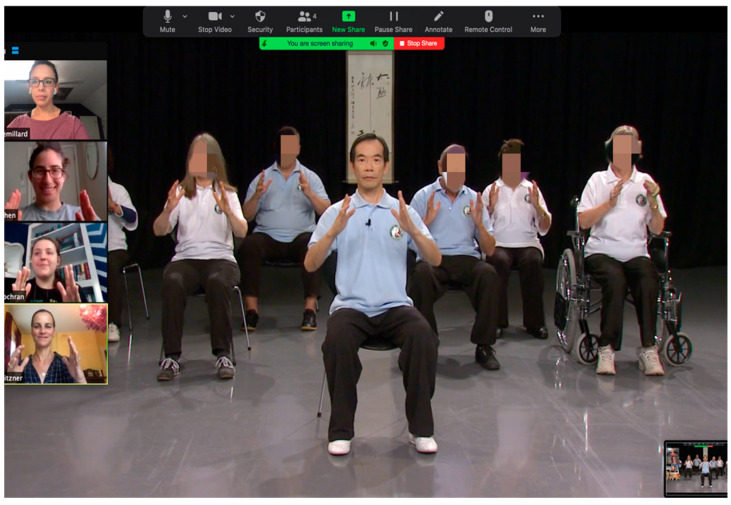
Study staff practicing Tai Chi for Arthritis and Fall Prevention (TCAFP) along with digital lesson.

Each 60 min class began with a technology check period (up to 10 min) to assist participants with technology troubleshooting or training. Next, participants engaged in moderated social time (10 min) answering discussion questions about themselves (e.g., hobbies, where they are from) or exercise (e.g., goals, challenges; see Figure 1 for a screenshot during social time). Then, participants followed pre-recorded tai chi lessons (approximately 30 min) that were broadcast from the Tai Chi for Health Institute online portal (see Figure 2). Each lesson consisted of warm-up exercises, learning a new movement, performing a sequence of previously learned movements, and cool-down exercises. Following the tai chi lesson, the class facilitator led another 10 min of moderated social time, which centered on a discussion about tai chi movements and principles. Participants were encouraged, but not required, to keep their cameras on during the exercise portion for safety monitoring. Our adaptation committee deemed this was appropriate as risks were similar to a person exercising along with the TCAFP program video at home by themselves, in a non-group setting.

### 2.4. Intervention Fidelity

We developed a detailed manual of procedures and training protocol to ensure both standardization of the administration of the assessment measures and treatment fidelity for each cohort. All researchers were thoroughly trained to administer assessments and to perform relevant roles (e.g., class facilitator, technical moderator) and used structured scripts for participant interactions (e.g., screening, orientation, assessments, classes). The technical moderator completed a fidelity checklist for each class to ensure all intervention components were delivered. The checklist was reviewed with the facilitator for accuracy immediately following each class. Any discrepancies were resolved through discussion until agreement was reached. Weekly team meetings were held to discuss each week’s classes and address any issues. We used a structured adaptation process to make any necessary adjustments to the intervention during the trial, guided by the adaptation committee described above. Further details of the study protocol and adaptations were described by Remillard et al. [60].

### 2.5. Outcome Measures

The primary outcome measures were self-reported physical activity (Physical Activity Scale for Individuals with Physical Disabilities [63]) and social connectedness (UCLA Loneliness Scale, ULS-8 [64]; modified Social Disconnectedness Questionnaire [65,66,67]). Secondary measures included exercise self-efficacy (Exercise Self-Efficacy Scale [68]), falls efficacy [69], fear of falling (adapted from [70,71]), depression (PROMIS Emotional Distress Depression Short Form [72]), quality of life (Kemp Quality-of-Life single-item scale [73]), and pain (PROMIS Pain Interference—Short Form [74]). Other outcome measures were technology acceptance (pre- and post-intervention only; adapted from the Technology Acceptance Model [75,76,77]) and class satisfaction (post-intervention only; Physical Activity Class Satisfaction Questionnaire [78]). Sociodemographic data were collected in the pre-intervention assessment. Detailed information on assessments, including measure modifications, is presented in prior publications [60,79]. All data is publicly available [80].

### 2.6. Statistical Analyses

Missing data were assessed at three levels (variable, case, and pattern), and the missing-at-random (MAR) assumption underlying the linear mixed models was probed empirically. Per-timepoint missingness was modest (0–10%): of the 60 enrolled participants, 51 had complete data on all primary and secondary outcomes across all three timepoints, and the 1-month follow-up was completed by 55 of 60 (91.7% retention), with predominantly monotone dropout. For multi-item composite scales, the 80% prorating rule was applied; PROMIS Pain Interference 4a required all four items. Linear mixed models with REML and an unstructured repeated covariance accommodate missing data under MAR without imputation. To test MAR, Welch’s *t*-tests compared 1-month follow-up completers (*n* = 55) and dropouts (*n* = 5) on baseline outcomes: no significant differences emerged on the psychosocial outcomes or the PASIPD leisure total (*p*s > 0.15), but dropouts reported lower baseline values on the walking/wheeling, moderate-sport, and strenuous-sport activity components (*t*s = 2.7–4.1, *p*s = 0.009 to <0.001). The differences on activity components suggest dropout was related to baseline activity level, and that mixed models remain valid under MAR conditional on observed data.

Outcome scores were screened for distributional properties via Shapiro–Wilk tests at each timepoint, and for residual normality of the fitted linear mixed models. The PASIPD leisure total and its five activity components were log+1 transformed to address marked right-skew on the raw scale (Shapiro–Wilk *W*s = 0.58–0.85, all *p*s < 0.001). Residual diagnostics on the analysis scale showed approximate normality for PASIPD log+1 and the social connectedness measures and modest residual deviations (heavy tails) for Falls Efficacy, the PROMIS T-scores, Quality of Life, and the Fear of Falling outcomes; for these, median and interquartile range are also reported in Table 1 alongside mean and standard deviation.

Linear mixed models with restricted maximum-likelihood (REML) estimation, unstructured repeated covariance, and Satterthwaite-approximated denominator degrees of freedom were fit to each outcome. For the Social Connectedness subscale analyses, likelihood-ratio χ^2^ tests were used in place of the standard LMM framework. Time (baseline, post-intervention, and one-month follow-up) was the sole fixed effect.

All inferential tests used α = 0.05, two-tailed. Omnibus time effects were tested with Type III F-tests; pairwise contrasts among the three timepoints were Bonferroni-adjusted within each outcome. Estimated marginal means and 95% confidence intervals were computed on the analysis scale and back-transformed via exp(EMM) – 1 for PASIPD outcomes to permit interpretation in MET hours per week. Intraclass correlation coefficients (ICCs) were computed from null models (random intercept only) to quantify the proportion of total variance attributable to between-person differences; descriptive statistics, ICCs, and LMM results for all outcomes are reported in Table 1.

Effect sizes are reported as partial η^2^ (omnibus time effect) and Cohen’s *d_z* (significant pairwise contrasts); 95% confidence intervals were computed for *d_z* [81]. Intraclass correlation coefficients (ICCs) represent the proportion of total outcome variance attributable to between-person differences (ICC(1); [82]) and were interpreted using common multilevel-modeling conventions [83,84]: <0.10 = within-person variation predominates, 0.10–0.30 = moderate share of between-person variance, and >0.30 = substantial clustering.

## 3. Results

### 3.1. Sociodemographic and Health Characteristics

Participants were mostly female (73%) with high levels of education (84% with a Bachelor’s degree or higher). Race was primarily white (80%), with 18% black or African American and 2% who did not wish to answer. Most participants lived with someone else (67%), whereas 33% reported living alone. Most participants reported using mobility aids (e.g., grab bars, cane, walker, wheelchair). All participants reported having at least one health condition, the most common being arthritis (60% of the sample). Additional details about participant characteristics and a CONSORT diagram are presented in Remillard et al. [60]. Notably, the sample was predominantly White and highly educated, which should be considered when interpreting these findings and their applicability to more diverse populations.

### 3.2. Technology Experience

In the baseline questionnaire, we asked participants to indicate their technology experience for 10 technologies related to the intervention. The majority of participants reported that they did not know what the technologies were, they did not use them, or they only used them once. The exceptions were FaceTime (70% reported using it occasionally or frequently) and Zoom (95% reported using it occasionally or frequently).

### 3.3. Feasibility

Feasibility was reflected by high rates of retention, low attrition, high adherence, technology use, program fidelity, program adaptation to the population, and safety. Just over 13% of participants (eight) were lost to attrition at the post-intervention timepoint. The reasons provided included: vision and hearing difficulties, pain and other health issues, scheduling conflicts/too busy, and disinterest in class content. There was also high adherence during the intervention. Altogether, participants attended 79.48% of the program’s 16 classes; over half of the participants attended 15 or more classes, and 12 participants attended all 16 classes. The attendance rate was similar to prior remote exercise programs. Wu et al. [33] reported 69% mean attendance in a telehealth tai chi trial for older adults, and Toledano-Shubi et al. [1] reported 79.7% mean attendance across 28 videoconference exercise interventions. Therefore, our adherence rate falls within the range observed in comparable programs. Mean participant attendance was 12.71 (*SD* = 4.26) classes. Not only did most participants adhere to the intervention sessions, but some also reported practicing outside of the classes. In each post-class survey, participants were asked how often they practiced tai chi on their own, outside of class, since the previous class. Out of 617 total responses, 12% stated they practiced more than three times, 21% practiced twice, 35% once, and 30% never (2% did not respond).

Furthermore, participants reported high rates of intention to adopt tai chi in the post-intervention interview, with 77% responding yes to “Do you plan to continue practicing tai chi?” (9% reported no; for 14% the response was not clear; *n* = 57). A month later, in the 1-month follow-up interview, 60% reported practicing tai chi in the past month (21.2% less than 1 day/week, 51.5% 1–2 days/week, 18.2% 3–4 days/week, 9.1% 5–7 days/week; *n* = 55). When asked if they would like to use Zoom to participate in group tai chi classes in the future, 65.5% selected “agree” or “strongly agree” (*n* = 55).

Program fidelity was evaluated with a fidelity checklist that was completed during each class by the technical moderator. The checklist included the following primary fidelity items: (1) attendance was taken, (2) the facilitator performed a technology check (i.e., confirmed all participants could see and hear), (3) participants were instructed how to get help from the technical moderator if needed, (4) the appropriate tai chi lesson was provided, and (5) the facilitator led social time with the appropriate discussion question. The checklist was fully completed 98.75% of the time over the course of the 16 classes. Specifically, only one element was not completed (i.e., in one class, participants were not instructed how to get help from the technical moderator). The trial also confirmed that the *TCAFP* program could feasibly be adapted to those aging with mobility disabilities, including using visual imagery for those with severe mobility limitations. With respect to safety, no participants reported adverse events during the study.

### 3.4. Outcomes

#### 3.4.1. Primary Outcomes

**Physical activity.** Leisure physical activity was assessed using the Physical Activity Scale for Individuals with Physical Disabilities (PASIPD [63]). All point and interval estimates in the PASIPD rows of Table 1 are reported on the back-transformed (MET h/wk) scale; F and *p* values reflect tests conducted on the analysis (log+1) scale. The linear mixed model indicated a significant effect of time. Pairwise comparisons revealed a significant increase in leisure physical activity from baseline to post-intervention, *p* < 0.001. Activity levels at the one-month follow-up did not differ significantly from baseline (*p* = 0.092). The significant increase from baseline to post-intervention, followed by a partial decline at follow-up (post-intervention vs. follow-up: *p* = 0.207), suggests that participation in the tai chi program was associated with increased leisure physical activity that was partially maintained one month after the intervention ended.

**Figure 3:**
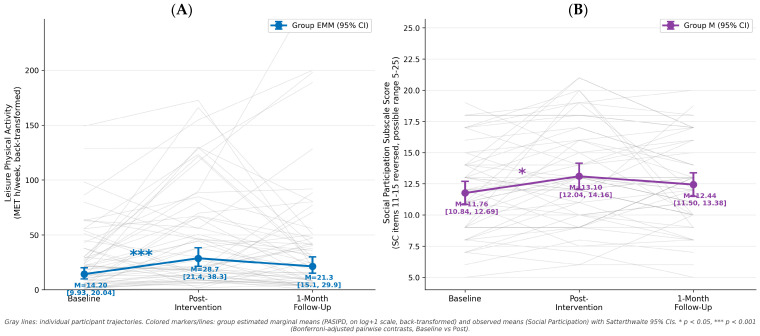
Individual and group trajectories of leisure physical activity (PASIPD; back-transformed estimated marginal means in MET h/week (**A**)); and Social participation subscale scores (observed means (**B**)); across the three timepoints. Gray lines show individual participant trajectories; colored markers and error bars show group means with 95% confidence intervals (PASIPD CIs derived on the log+1 scale and back-transformed, asymmetric on the raw scale). Asterisks denote a significant baseline-to-post change: * *p* < 0.05, *** *p* < 0.001.

To characterize the distribution of leisure physical activity across domains, supplemental analyses examined each of the five PASIPD leisure activities separately using log+1 transformed scores: walking or wheeling outside (PA1), light sport or recreational activities (PA2), moderate sport or recreational activities (PA3), strenuous sport or recreational activities (PA4), and muscle strengthening or endurance activities (PA5). Walking and wheeling outside (PA1) showed a significant effect of time, with a significant increase from baseline to post-intervention, *p* = 0.002, followed by a partial decline at follow-up (post-intervention vs. follow-up: *p* = 0.69). Light sport and recreational activities (PA2) also showed a significant effect of time, with a significant increase from baseline to post-intervention, *p* < 0.001, followed by a significant decline at follow-up, *p* = 0.009. Moderate sport (PA3) showed a significant effect of time, with a significant increase from baseline to follow-up, *p* = 0.02; the baseline to post-intervention and post-intervention to follow-up changes were not significant, *p* = 0.35. Strenuous sport (PA4) did not show a significant effect of time. Muscle strengthening (PA5) showed a significant effect of time, with a significant increase from baseline to post-intervention, *p* = 0.035; activity levels returned toward baseline at follow-up, *p* = 1. See Figure 3 for trajectories of leisure physical activity (PASIPD; back-transformed estimated marginal means in MET h/week) across the three timepoints.

**Social connectedness.** Social connectedness was assessed using an adapted version of the Social Connectedness Questionnaire [65,66,67], a 15-item self-report measure (possible range: 15–67; higher scores indicate greater connectedness). The linear mixed model did not reveal a significant effect of time, *F*(2, 53.5) = 2.43, *p* = 0.098. The omnibus test of overall social connectedness did not reach significance, and the pre-to-post contrast was not significant after Bonferroni adjustment (*p* = 0.115). Although the unadjusted contrast was suggestive (*p* = 0.038), this should be interpreted cautiously, particularly after correction for multiple comparisons. In contrast, the Social Participation subscale (items 11–15) did show a significant increase from baseline to post-intervention, as reported below.

To further explore patterns within the Social Connectedness measure, subscale-level LMMs were conducted on the four constituent dimensions: Network Characteristics/Size (items 1–4), Social Support (items 5–7, reverse-coded), Loneliness and Isolation (items 8–10), and Social Participation (items 11–15, reverse-coded). Social Participation was the only subscale to show a significant effect of time, *p* = 0.014. Bonferroni-corrected pairwise comparisons revealed a significant increase from baseline to post-intervention, *p* = 0.010. Scores at follow-up did not differ significantly from baseline (*p* = 0.543) or post-intervention (*p* = 0.350). Network Size (*p* = 0.518), Social Support (*p* = 0.743), and Loneliness and Isolation (*p* = 0.491) showed no significant changes across timepoints. These results suggest that the observed trend in overall social connectedness was primarily driven by improvements in social participation. Descriptive statistics and LMM results for all four Social Connectedness subscales are presented in Table 2. See Figure 3 for trajectories of the Social Participation subscale scores across the three timepoints.

**Loneliness.** Loneliness was assessed using the UCLA Loneliness Scale—8-item version (ULS-8 [64]; possible range: 8–32; higher scores indicate greater loneliness). The linear mixed model did not reveal a significant effect of time. The mean scores across all timepoints fell in the lower range of the scale (14.9–15.3 out of a possible range of 8–32), indicating relatively low levels of loneliness at baseline that remained stable throughout the intervention. This pattern likely reflects a floor effect in which participants entered the trial with already-low loneliness scores, which limited the intervention’s ability to produce detectable changes in loneliness.

#### 3.4.2. Secondary Outcomes

**Exercise self-efficacy.** Exercise self-efficacy was assessed using the Exercise Self-Efficacy Scale (ESES [68]; possible range: 10–40; higher scores indicate greater confidence). The linear mixed model did not reveal a significant effect of time. Means showed a small positive trend in the expected direction.

**Falls efficacy.** Falls efficacy was assessed using the Falls Efficacy Scale (FES [69]; possible range: 10–100; lower scores indicate greater confidence). The linear mixed model did not reveal a significant effect of time.

**Fear of falling—severity.** Fear of falling severity was assessed with a three-part item [70,71]. Participants first indicated whether they experienced fear of falling (yes/no); those who responded affirmatively rated the degree of fear (1 = somewhat, 2 = fairly, 3 = very fearful). A composite severity variable was coded 0 for no fear and 1–3 for graded fear. The linear mixed model indicated no significant effect of time.

**Fear of falling—activity restriction.** Activity restriction due to fear of falling was assessed by asking whether fear of falling had caused participants to limit their activities (0 = No, 1 = Yes). Results indicated no significant effect of time, *F*(2, 52.6) = 2.05, *p* = 0.139.

**Emotional distress.** Emotional distress was assessed using the PROMIS Emotional Distress—Depression—Short Form 8b [72]. Scores were converted to T-scores (population *M* = 50, *SD* = 10; range 37.1–81.1; higher scores indicate greater distress). The linear mixed model did not reveal a significant effect of time.

**Quality of life.** Quality of life was assessed using the Kemp Quality-of-Life single-item scale [73] (range: 1–7; higher scores indicate better quality of life). The linear mixed model did not reveal a significant effect of time.

**Pain interference.** Pain interference was assessed using the PROMIS Pain Interference—Short Form 4a [74]. Scores were converted to T-scores (range 41.6–75.6; higher scores indicate greater pain interference). The linear mixed model did not reveal a significant effect of time, *F*(2, 54.4) = 0.29, *p* = 0.748. PROMIS Pain Interference T-scores from 20 to 55 are considered within normal limits; 55–60 is mild, 60–70 is moderate, and 70 and over is severe. A score change of 2–3 points has been reported as a clinically meaningful difference. Mean scores across all timepoints fell in the mild range of pain interference (T-scores 58.0–58.9).

Descriptive statistics, ICCs, and full LMM results for all primary and secondary outcomes are presented in Table 1.

#### 3.4.3. Technology Experience and Class Satisfaction

**Figure 4:**
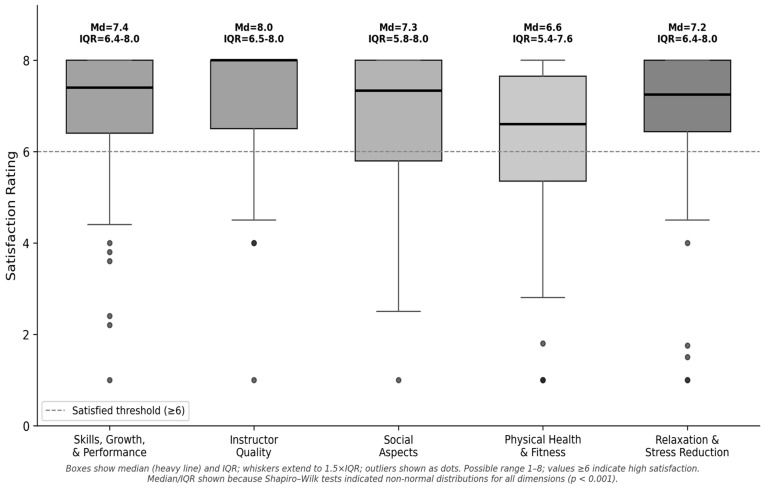
Class satisfaction ratings by dimension from the post-intervention questionnaire (N = 56). Boxes show the median and interquartile range; whiskers extend to 1.5× IQR and points are outliers. A boxplot is presented rather than mean ± SD because the ratings were non-normally distributed (Shapiro–Wilk *p* < 0.001 for all dimensions).

**Class satisfaction.** In the post-intervention questionnaire, participants rated their satisfaction with the program across five dimensions: skills, growth, and performance; instructor quality; social aspects; physical health and fitness; and relaxation and stress reduction. Ratings were on a scale from 1 (Not Satisfied) to 8 (Very Satisfied). Mean ratings were 6 or higher for all dimensions: instructor quality was rated highest (*M* = 7.12), followed by skills, growth, and performance (*M* = 6.74), social aspects (M = 6.69), relaxation and stress reduction (*M* = 6.66), and physical health and fitness (*M* = 6.12). See Figure 4 for mean satisfaction ratings by dimension (*n* = 56). Given the mean satisfaction ratings fell near the upper range of the scale (*M* = 6.12–7.12 out of a possible range of 1–8), there is the potential for a ceiling effect. This may have limited the sensitivity of this measure for detecting meaningful variation in satisfaction across participants and program dimensions.

**Technology acceptance.** In the post-intervention questionnaire, participants rated their acceptance of using Zoom, in general, and using Zoom to participate in group exercise classes, more specifically.

**Figure 5:**
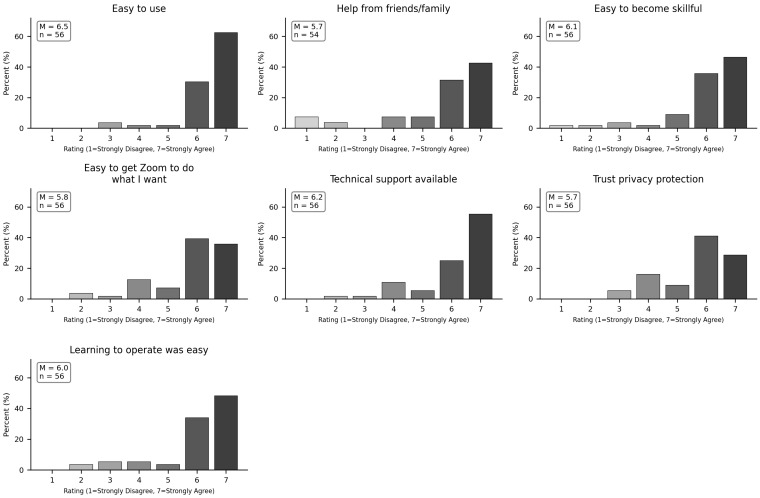
Post-intervention histograms for Zoom ease-of-use items.

Regarding using Zoom in general, participants overwhelmingly rated Zoom as easy to use (*n* = 56). Across the seven ease-of-use items (excluding security concerns, which was reverse-scored), mean ratings ranged from 5.71 to 6.46 on a 7-point scale (1 = Strongly Disagree, 7 = Strongly Agree). The highest-rated item was “I found Zoom easy to use” and the lowest was “I trusted in the ability of Zoom to protect my privacy”. Response distributions were heavily skewed toward agreement, with the majority of participants selecting 6 or 7 on most items (see Figure 5).

Regarding Zoom usefulness for exercise (*n* = 56), participants rated Zoom highly for facilitating group exercise participation. The highest-rated item was “Using Zoom made it easier for me to participate in group exercise classes” followed by “Enhanced my ability to engage in physical activity”, “Increased my daily physical activity”, and “Improved my daily life”.

**Figure 6:**
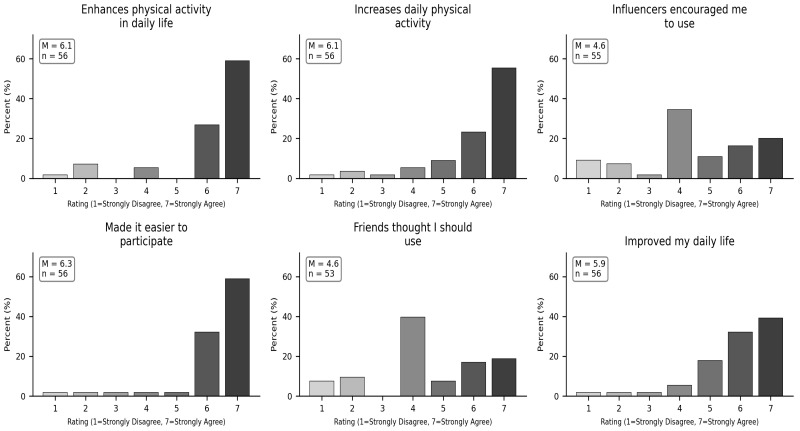
Post-intervention histograms for Zoom usefulness-for-exercise items.

Items reflecting social influence were rated lower than those of usefulness: “People who influence my behavior encouraged me to use Zoom” and “My friends thought that I should use Zoom”, suggesting that participants’ adoption was more internally than externally motivated (see Figure 6).

**Figure 7:**
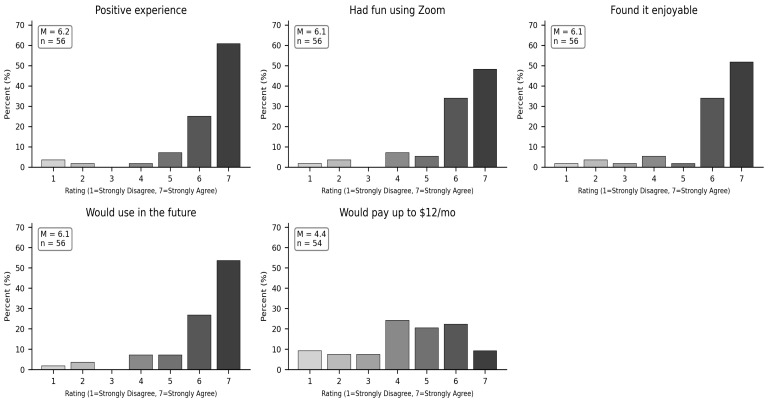
Post-intervention histograms for enjoyment and intent to adopt items.

Participants also reported high levels of enjoyment using Zoom for group exercise (*n* = 56). Mean ratings for the three enjoyment items ranged from 6.05 to 6.25: “I found using Zoom to participate in group exercise classes to be a positive experience”, “I had fun using Zoom to participate in group exercise classes” and “I found using Zoom to participate in group exercise classes to be enjoyable”. Response distributions were again heavily skewed toward agreement (see Figure 7).

Regarding intent to adopt, when asked whether they would plan on using Zoom to participate in group exercise classes in the future, most participants expressed agreement (*M* = 6.09). A total of 80.4% of participants selected “agree” or “strongly agree” (26.8% at 6, 53.6% at 7). These findings are consistent with the feasibility results, in which 77% of participants said they planned to continue tai chi during the post-intervention interview.

Participants’ positive attitudes toward Zoom were reflected in interviews. The following participant quotes are from post-intervention interviews and are presented as illustrative examples only. One stated that Zoom facilitated their participation, saying, *“Being that I have a big mobility issue, it’s like Zoom was invented for me. It allows me to participate in the outside world.”* And another shared that they felt the class felt inclusive of differing abilities, stating, *“It was sometimes reassuring to see…not everybody was perfect at this.”* Another spoke of the accessibility of Zoom but also the accountability the intervention afforded, saying, *“I loved being on Zoom, because it just made it a little bit more accessible, also made me accountable, because I had it scheduled.”*

It is important to note that despite high ratings for ease of use, participants did report technical difficulties in the post-class survey. Technical issues included poor audio/video quality, such as broadcasting video playback errors and inconsistent video streaming, unstable audio, non-synchronous audio, and low volume. They reported challenges launching Zoom, navigating Zoom features, and unstable internet. They also reported hardware issues, such as camera/microphone/speaker errors and broken equipment.

## 4. Discussion

People aging with mobility disabilities experience a variety of barriers to participating in wellness interventions in-person. To meet this need, we translated the evidence-based *TCAFP* program to a virtual format, made modifications to accommodate physical limitations (e.g., utilized a seated version, included visualization guidance), and added a social component. This single-arm clinical trial found significant pre–post changes in physical activity and social participation for adults aging with mobility disabilities from participating in the 8-week *TechSAge Tele Tai Chi* program. The pre–post changes were partially (but not significantly) sustained at 1-month follow-up for physical activity only. The trial also demonstrated the feasibility, acceptability, satisfaction, and enjoyment of a virtual tai chi class for adults aging with mobility disabilities.

Significant physical activity benefits were found comparing baseline to post-intervention measures and approached significance when comparing baseline to 1-month follow-up measures. Specifically, leisure physical activity as measured by the PASIPD significantly increased from baseline to post-intervention. The baseline-to-post increase corresponded to a medium effect (Cohen’s *d_z* = 0.54, 95% CI [0.26, 0.82]). Activity levels at one-month follow-up did not differ significantly from baseline, suggesting only partial maintenance of gains. Participants were given a free 3-month membership to the virtual *TCAFP* program through the Tai Chi for Health Institute. In the 1-month assessment, 60% reported practicing tai chi after the intervention ended, which could explain the partial maintenance of the physical activity changes from post-intervention to 1-month follow-up. The subscale analysis showed significant increases in walking or wheeling outside, light sport and recreational activities, moderate sport, and muscle strengthening, but not strenuous sport. These findings are not surprising, particularly that light recreational activity (which would reflect tai chi practice) drove much of the overall physical activity improvement, and suggest the physical activity increases were more domain-specific than an overall improvement in all aspects of physical activity. The magnitude of the physical activity improvement (baseline mean of 14.2 MET h/week to post-intervention mean of 28.7 MET h/week) represents almost a doubling of leisure physical activity. As context, the current physical activity guidelines recommend that adults with disabilities accumulate at least 150 min per week of moderate-intensity aerobic activity (~7.5–10 MET h/week for moderate activities [85]). Achieving this level may have meaningful implications for cardiometabolic health and functional independence, though direct clinical benefits remain to be confirmed through objective measurement and controlled trials.

Placing the magnitude of the physical activity improvement in context of the literature is complicated by differing PASIPD scoring conventions: we expressed leisure activity as a weekly total (MET h/week), whereas prior PASIPD studies report a daily average (MET h/day). Converting our scores to the daily metric, leisure physical activity rose from approximately 2.0 MET h/day at baseline to 4.1 MET h/day post-intervention (estimated marginal means; observed means 4.0 and 7.0 MET h/day). These levels are below those reported for other samples of adults with disabilities or chronic conditions assessed with the PASIPD: community-dwelling older adults (≥65 years) with multiple chronic conditions averaged 11.0 MET h/day [86], adults with physical disabilities attending a sports exhibition averaged 16.7 MET h/day [87], and community-dwelling persons with multiple sclerosis averaged 15.5–18.0 MET h/day at baseline [88]. The comparatively low activity of our participants is consistent with its defining characteristics of long-term, often severe mobility disability in older adulthood and a seated program format. Notably, none of these comparison samples received a tai chi intervention and none match our population precisely; the closest analogues are the multiple sclerosis education program of Feys et al. [88], in which the subgroup with less disability increased PASIPD by roughly 5–6 MET h/day, and the observational disability cohort of Nettleton et al. [87], in which PASIPD did not change over six months. Relative to these studies, the increase from baseline to post-intervention (close to doubling of leisure activity by estimated marginal means and almost a 75% rise in observed means) suggests the TechSAge Tele Tai Chi program meaningfully increased activity even though absolute levels remained below those of less mobility-limited groups. These findings add to the growing evidence base [37,43,54,89] showing that tai chi is feasible and safe for older adults with a wide range of abilities, including those with mobility disabilities and significant limitations (e.g., those unable to walk). This study also contributes to the more limited literature on the benefits of seated tai chi, which relies primarily on upper-body movements [90].

Overall measures of social connectedness and loneliness did not change significantly across timepoints. Notably, baseline scores indicated that participants were not experiencing high levels of loneliness or social disconnection before the intervention (i.e., floor effects). This may help explain the lack of significant change, as scores were already in a relatively favorable range, leaving limited room for improvement. Intraclass correlation coefficients were high for social connectedness and loneliness, indicating strong individual stability over time. The social component involved 20 min of moderated group discussion per session (10 min before and 10 min after the tai chi lesson). Although participants rated social aspects highly, the absence of significant change in overall social connectedness raises the question of whether this duration was sufficient to influence constructs such as loneliness, social support, and network size. Longer and perhaps more naturalistic social interactions may be needed to produce detectable change on these dimensions. While three of the four social connectedness subscales did not show improvement, participants did report significantly higher social participation rates at post-intervention as compared to baseline. It is possible that social participation is more sensitive to change, whereas the other subscales (i.e., network size, social support, loneliness and isolation) require more time or different types of discussion topics to improve. The social participation pre–post improvement effect did not remain when comparing pre-intervention to 1-month follow-up measures. Unlike the tai chi component, which participants received extended resources to continue practicing, no such formal continuation of the social aspects of the intervention was provided. This may help explain why increases in social participation were not maintained at the 1-month follow-up assessment. There were some instances in which participants asked to share contact information with each other, but this was not the norm. Despite the overall social connectedness factor not changing significantly across timepoints, it is possible that the social context of the group class contributed to the high adherence rates and enjoyment ratings. Group exercise can lend a sense of belonging [25], and this might have been stronger because the participants had in common that they were aging with mobility disabilities.

None of the secondary measures changed significantly when comparing baseline, post-, and 1-month follow-up timepoints. One explanation is that participants’ baseline scores were in favorable ranges, limiting room for improvement. They reported relatively high scores for quality of life, exercise self-efficacy, and falls efficacy, and low scores for pain and depression. This pattern could reflect a self-selection bias, in that participants who enrolled in the trial were more motivated and focused on health, resulting in a sample that was healthier than the broader population of adults aging with mobility disabilities. It is possible that the secondary outcome measures we chose were not sensitive enough to detect small changes in this higher-functioning sample. It is also possible that the intervention duration was not long enough to produce measurable changes in the secondary outcomes. Nevertheless, the lack of declines over time provides additional evidence that the intervention was safe and that participants were able to maintain their quality of life, exercise self-efficacy, falls efficacy, pain, and depression status from the baseline timepoint to the 1-month follow-up timepoint. Of note is that activity restriction related to fear of falling trended upward, suggesting participants were more avoidant comparing baseline to 1-month follow-up. While not statistically significant, one speculation is that this trend reflected increased awareness of fall risk following participation in the fall-prevention-focused program. However, this interpretation is purely exploratory and cannot be confirmed without additional data, such as qualitative accounts or a control comparison group.

Feasibility was demonstrated through strong retention and adherence, low attrition, high technology acceptance ratings, high program fidelity, successful adaptation of the program to the target population, and the absence of any adverse events or injuries. The intervention sessions were highly attended, and most participants completed the 8-week intervention. Participants reported missing sessions for the following reasons: away from home or out of town, schedule conflict, personal emergency, memory slip, or holiday. Some participants reported practicing tai chi on their own during the intervention, as well as during the post-intervention and 1-month follow-up assessments. All participants were able to use Zoom successfully to participate in the intervention and most reported that it was easy to use. Several factors likely contributed to their success. Most had some prior experience using Zoom (35% used it occasionally, 60% used it frequently). We provided technical support in the form of written instructions, group training during the technology check time that was built into the beginning of every session, and individual troubleshooting with the technical moderator during the technology check time if needed. Participants did experience some technical challenges, although most technical issues were resolved.

The intervention had high fidelity that was supported by comprehensive staff training, manuals, and scripts. We streamed the tai chi lessons from the Tai Chi for Health Institute online portal, thereby ensuring all participants received the same exercise instruction. The specific tai chi lessons we provided were from the *TCAFP* program, which was designed to be appropriate for a wide range of abilities and able to be performed from a seated position. This made the program especially easy to adapt to our target population of people aging with mobility disabilities. Lastly, the intervention was feasible in terms of optimizing safety. We developed remote safety protocols, including having participants use a sturdy chair or locked wheelchair, provide emergency contact information, and have a phone within reach during sessions, and no participants experienced an adverse event during the trial.

The intervention was well-received with respect to acceptability, as well. Participants’ ratings for satisfaction, ease of use, enjoyment, and perceived physical and social benefits were all high. Satisfaction was highly rated across several dimensions, including skills, growth and performance, physical health, relaxation, and social environment. Participants reported that Zoom was easy to use and useful for participating in a group exercise class. They also showed high rates of intention to adopt. Furthermore, participants gave the program high ratings for enjoyment.

Participants did experience technical difficulties, yet satisfaction and enjoyment ratings remained high despite these challenges. One possible interpretation that is consistent with prior research showing that older adults make benefit-driven technology adoption decisions [91] is that participants found the benefits to outweigh the inconvenience of technical issues. However, this interpretation is inferential, and future research is needed to directly assess how technical barriers affect program satisfaction and engagement. Qualitative findings suggest that fostering group social support promotes a more enjoyable exercise experience [92]. The social dynamic is significant as older adults often prioritize enjoyability, sociability, affordability, accessibility, and flexibility over health benefits when evaluating an exercise class [93]. These social factors also appear to drive longer-term engagement. A systematic review identified social connectedness, perceived benefits, program design, empowering effects, and instructor behavior as key themes for adherence to community-based group exercise programs [94].

Older adults, in general, have less technology experience than younger adults, although the gap is narrowing [95]. The *TechSAge Tele Tai Chi* intervention was designed to support the success of participants’ interactions with the technology aspects of the intervention and be accessible to those with less technology experience. The high rating for ease of use suggests we were relatively successful and may be attributed to multiple aspects of the intervention. For one, those with more technology experience may have been more likely to join the study (i.e., self-selection bias). Having access to a computer or tablet and internet access were inclusion criteria, which would have excluded those with especially minimal or no technology experience. Moreover, we provided multiple aspects of technology support, including training during the individual orientation, support materials, and access to troubleshooting support from the technical moderator.

The *TechSAge Tele Tai Chi* intervention was also accessible to adults aging with mobility disabilities in terms of physical ability. These findings add to the previous literature on the accessibility of tai chi for people with a range of health conditions and physical abilities [37,54,89]. Some of the participants in the study had significant disabilities, such as being unable to walk or having limb restrictions (e.g., limited reach, unable to move). With the guidance of our adaptation committee, we were able to make adaptations that enabled each person in the study to participate for the entirety of the program. For those with significant physical limitations, that included visualizing the movement rather than performing it physically. They were also provided information about the research on mental imagery, specifically that motor imagery activates neurons similar to those activated by physical movements [61]. This adaptation improved accessibility for participants with severely limited movement and aligns with rehabilitation research demonstrating the benefits of motor imagery [96].

Despite the strengths of this study (e.g., targeted underserved population, sufficient training and technical support, adaptation of an evidence-based program), there are limitations. The single-arm design without a control group prevents causal conclusions. In the absence of a control group, it is not possible to determine whether the observed increase in physical activity was specifically attributable to the intervention alone or whether other factors contributed, such as increased self-monitoring, attention effects, or variation in activity levels unrelated to the intervention. In addition, use of self-reported outcomes may have introduced recall bias and social desirability effects. Participants were aware of the study goals and their own participation in the intervention, which may have positively inflated self-reports. Because participants enrolled in a wellness program they anticipated would be beneficial, expectancy effects may have contributed to the pre–post changes observed in self-reported physical activity and social participation, as well as to the high acceptability, satisfaction, and enjoyment ratings. Knowledge that their behavior was being observed and assessed at multiple timepoints may likewise have prompted increased activity or more favorable reporting independent of the intervention’s active components (i.e., a Hawthorne effect). The repeated assessment schedule and the use of a camera during the exercise may have heightened this awareness. These effects cannot be disentangled from the intervention effects in a single-arm design, and they reinforce the need for a randomized controlled trial with a waitlist, attention-matched, or active control condition to establish how much of the observed benefit reflects the intervention itself rather than expectancy. A future trial should also incorporate objective performance measures in addition to self-report measures. Such a trial should be powered based on the effect sizes observed here (e.g., F = 9.30 for physical activity) and should incorporate objective measures, particularly for physical performance, as well as subjective measurements of visualization use.

The convenience sample has limited generalizability. It was predominantly White and highly educated, and likely over-represents motivated, digitally capable participants. People with disabilities from underrepresented racial and ethnic groups face compounding barriers to telewellness participation, including lower household broadband and device access, more limited prior experience with videoconferencing, and poorer self-rated health, all of which are relevant to whether the program would translate to a more diverse sample [97,98]. Including a larger and more demographically diverse sample (e.g., oversampling underrepresented racial/ethnic groups and limited prior technology experience) would provide more generalizable results and can evaluate whether additional onboarding or technology support is needed for equitable access.

Causes of long-term mobility disability were not strategically assessed or sampled. Participants represent a variety of health conditions (e.g., arthritis, multiple sclerosis, diabetes, post-polio syndrome) that likely contributed to their mobility challenges [60]. As such, the sample represents a wide range of lower and upper body functional mobility limitations and use of mobility aids. The heterogeneity in disability cause and the resulting variability in disability level limit conclusions about which subgroups benefit most and reduce generalizability to specific disability populations. At the same time, this diversity may be considered a strength, as the population aging with mobility disability is itself diverse in capabilities and support needs. In addition, we specifically designed the *TechSAge Tele Tai Chi* program to be accessible to those with mobility disabilities, which limits the accessibility implications of the findings to this specific disability population. Future work should also explore adaptations for individuals with vision and hearing disabilities.

It is possible that the sample size was not sufficient to detect a significant change in social connectedness. With 60 participants, the study may have been underpowered to detect small-to-moderate effects on social connectedness; indeed, the omnibus test approached but did not reach significance, and baseline scores were already in a relatively favorable range, creating potential ceiling effects that would require a larger sample to overcome. A post hoc power analysis indicated that approximately 100 participants would be needed to detect a medium effect size (*f* = 0.25) with 80% power for repeated-measures comparisons across three timepoints. It is also conceivable that the 1-month follow-up was too short to assess long-term maintenance.

We included 30 min of tai chi to keep the sessions at an hour and allow for 10 min of technology time and 20 min of social time. It may be that the tai chi exposure time was too short to find significant effects in the secondary measures. In the Chen et al. [54] meta-analysis, tai chi exposure times ranged from 12 to 144 h (Mean = 43.13 h, Median = 36 h, and Mode = 24 h). In the Huang et al. [37] meta-analysis, tai chi exposure time ranged from 24 to 173.4 h (Mean = 93 h, Median = 104 h, Mode(s) 48 h and 173.4 h). Ours was relatively short at 8 h over 8 weeks. However, the 8 h dosage refers exclusively to supervised tai chi lesson time delivered during the 16 group sessions (~30 min per session) and does not include home practice. Across post-class survey responses, 68% indicated participants had practiced at least once outside of class since the previous session, suggesting total tai chi exposure was likely higher than supervised tai chi lesson time. Nevertheless, there have been 8-week programs that did find significant improvements [43]. The significant heterogeneity across research studies underscores the need for more research to identify the most effective dosage of tai chi [37,54]. In addition, it is important to note that the measures in this study were all self-report, which limits the interpretation of physical benefits. We do not know if participants had observable physical benefits from the intervention. However, it is possible given prior research showing improvements such as balance, flexibility, and lower limb strength [40,55,99].

A different type of social component (e.g., longer durations, different discussion topics, more naturalistic discussion) may produce greater benefits. Ideally, an evidence-based social intervention could be paired with the tai chi aspects of the program to be significantly impactful for physical and social wellness. Nevertheless, our findings suggest that a group virtual program is feasible for older adults (with appropriate training and support), and could potentially be translated to other wellness programs, such as other forms of physical activity, social programs, and financial literacy.

Interest in tai chi continues to grow, supported by mounting evidence that it is a safe, enjoyable, and efficacious light-to-moderate aerobic activity for older adults [37,54,89]. This study addresses a gap that is timely and practically relevant. Community-based organizations have delivered TCAFP since before the COVID-19 pandemic, yet many shifted to virtual delivery during the pandemic. To our knowledge, this is the first study to demonstrate that the virtual adaptation is feasible and can be implemented safely. In this sense, our findings provide initial validation for a mode of delivery that is already occurring in real-world community settings, rather than serving solely as a precursor to a larger trial. It is also innovative in that it shows that an accessible, virtual adaptation of the program can be implemented safely and is feasible and acceptable for older adults with mobility disabilities. It is inexpensive, with minimal space and equipment needed, making it particularly suitable for community-based and preventive health models [89]. TCAFP is already classified as a Title III-D Highest Tier Evidence-based Program by the Administration on Community Living and the CDC. A virtual delivery model could increase the accessibility and scalability of *TCAFP* because there are instances in which an in-person program is not possible. Some community-based organizations do not have access to in-person certified tai chi instructors (e.g., rural locations) and some do not have the funding to pay for instructors to travel to their organization. Moreover, some participants experience transportation barriers getting to in-person programs. Adults with mobility disabilities are especially impacted by such barriers. Providing a virtual opportunity to participate in a safe and effective physical activity program is a promising solution. Virtual formats may also facilitate the scaling of programs more efficiently (e.g., Area Agencies on Aging’s scaling programs to their affiliated senior centers). While the present results are promising, this trial did not assess deployment costs, training requirements for community organizations, digital literacy or equity barriers that may limit uptake in underserved communities. Deployment studies are needed to evaluate the feasibility, resource requirements, and cost-effectiveness of scaling *TechSAge Tele Tai Chi* before widespread implementation can be recommended. Moreover, future work is needed to evaluate the feasibility and effectiveness of different telewellness delivery models (e.g., live instruction, larger classes, hybrid classes with a mix of in-person and online participants).

There is a need to determine the long-term implications of tai chi interventions. The partial maintenance of physical activity gains in the present study at 1-month follow-up (M = 21.3 MET h/week) suggests continued access to the program may be necessary to sustain physical activity increases. Strategies to support long-term maintenance might include extended program access or peer practice groups. Long-term tai chi practitioners show sustained functional benefits [39,54,100], suggesting continued practice is beneficial. The Chen et al. [54] meta-analysis study found that the effectiveness of tai chi increased with the duration and frequency of exercise. Some studies have explored long-term tai chi practitioners. In a cross-sectional study, long-term tai chi “experts” performed better than age-matched tai chi naive controls and were statistically indistinguishable from young healthy adult controls for a range of functional physical performance tasks [100]. Other findings suggest that long-term tai chi training may benefit brain functioning and neural networking [101]. Research is needed to determine the frequency and duration needed for tai chi interventions to have longer-term benefits.

Although the social component of our intervention had limited benefit, research documenting the significant negative impacts of social isolation [7] highlights the need to continue this line of research. Future work should explore different types of social interaction, including longer durations, more naturalistic discussion, and activities that facilitate social connection between sessions, to determine which characteristics are most beneficial for improving social connectedness and reducing loneliness in this population. Many group exercise interventions do not measure social outcomes, leaving a significant gap in the literature. Social support has been linked to better health and functioning in populations with physical disabilities [19], suggesting that more targeted social interventions, both standalone and those embedded within exercise programs, warrant further investigation, particularly in virtual formats.

## 5. Conclusions

Adults aging with mobility disabilities face significant barriers to participating in evidence-based wellness programs in person, yet few interventions have been designed to meet their needs. The *TechSAge Tele Tai Chi* clinical trial translated an evidence-based tai chi program to a virtual and physically accessible format, with an added social component, to meet this need. This single-arm clinical trial found significant pre–post changes in physical activity and social participation for adults aging with mobility disabilities, extending the tai chi evidence base to adults aging with mobility disabilities, including those with severe physical limitations. The absence of declines in secondary outcomes supports the intervention’s safety. High rates of retention, adherence, fidelity, satisfaction, enjoyment, and intention to continue demonstrate that this population can successfully engage in and enjoy a virtual wellness program when provided with appropriate adaptations and support. These findings highlight the potential of virtual delivery models to expand access to evidence-based programs for those who face the greatest barriers to in-person participation and suggest that this approach could be extended to other wellness programs beyond tai chi. Future research should examine optimal dosage, long-term benefits, and more targeted social interventions. Beyond establishing a foundation for a future effectiveness trial, the findings from this study offer the first evidence that virtual delivery of TCAFP (a model already in practice) can be safe, feasible, and acceptable even for adults aging with mobility disabilities. Deployment studies are now needed to assess the feasibility and cost-effectiveness of scaling *TechSAge Tele Tai Chi* through community-based organizations such as senior centers, support groups, and senior living communities.

## Figures and Tables

**Table 1:** Primary and outcomes outcomes (N = 60).

**Outcome (Scale)—log+1 Analyzed; Reported Back-Transformed**	**Timepoint**	**n**	**M (EMM)**	**Median [Q1, Q3]**	**Min, Max**	**95% CI of EMM (Back-Transformed)**	**F (df1, df2)**	***p***	**Partial η^2^ (Cohen’s f)**
PASIPD leisure total (MET h/wk)	Baseline	60	14.2	21.2 [5.2, 37.9]	0.0, 148.9	[9.93, 20.04]	9.30 (2, 54.4)	<0.001	0.255 (0.58)
	Post	56	28.7	33.8 [13.0, 67.7]	1.6, 173.2	[21.37, 38.34]			
	Follow-up	55	21.3	26.9 [7.9, 48.9]	0.0, 272.1	[15.08, 29.95]			
PASIPD—walking/wheeling (MET h/wk)	Baseline	59	4.2	3.7 [1.6, 11.1]	0.0, 37.9	[2.88, 5.88]	6.87 (2, 54.4)	0.002	0.202 (0.50)
	Post	56	8.4	9.5 [3.7, 19.7]	0.0, 63.1	[6.03, 11.46]			
	Follow-up	54	6.6	4.7 [3.7, 18.9]	0.0, 63.1	[4.71, 9.20]			
PASIPD—light sport (MET h/wk)	Baseline	59	2.7	2.3 [0.0, 6.8]	0.0, 89.9	[1.64, 4.06]	14.63 (2, 54.9)	<0.0001	0.348 (0.73)
	Post	55	7.4	9.0 [5.2, 15.8]	0.0, 89.9	[5.34, 10.19]			
	Follow-up	53	4.0	5.2 [0.0, 13.4]	0.0, 54.1	[2.59, 5.93]			
PASIPD—moderate sport (MET h/wk)	Baseline	59	0.7	0.0 [0.0, 0.0]	0.0, 31.5	[0.31, 1.18]	4.25 (2, 54.9)	0.019	0.134 (0.39)
	Post	55	1.4	0.0 [0.0, 6.9]	0.0, 54.1	[0.62, 2.44]			
	Follow-up	55	1.8	0.0 [0.0, 10.5]	0.0, 180.3	[0.97, 3.12]			
PASIPD—strenuous (MET h/wk)	Baseline	59	0.5	0.0 [0.0, 0.0]	0.0, 134.6	[0.12, 0.87]	1.52 (2, 55.3)	0.228	0.052 (0.23)
	Post	56	0.8	0.0 [0.0, 0.4]	0.0, 78.8	[0.34, 1.44]			
	Follow-up	55	0.9	0.0 [0.0, 1.1]	0.0, 134.6	[0.35, 1.58]			
PASIPD—muscle strength (MET h/wk)	Baseline	60	1.7	0.0 [0.0, 9.6]	0.0, 49.4	[0.92, 2.75]	3.78 (2, 55.2)	0.029	0.120 (0.37)
	Post	56	3.2	4.1 [0.0, 9.6]	0.0, 99.5	[2.00, 5.02]			
	Follow-up	55	2.0	0.0 [0.0, 9.6]	0.0, 57.6	[1.13, 3.36]			
**Outcome (Scale)**	**Timepoint**	**n**	**M (SD)**	**Median [Q1, Q3]**	**Min, Max**	**95% CI of EMM**	**F (df1, df2)**	***p***	**Partial η^2^ (Cohen’s f)**
Social Connectedness (composite, 15–67)	Baseline	60	42.85 (8.10)	43.00 [38.00, 49.25]	18.00, 57.00	[40.75, 44.94]	2.43 (2, 53.5)	0.098	0.083 (0.30)
	Post	55	44.67 (7.61)	44.00 [39.00, 50.00]	28.00, 59.00	[42.08, 46.34]			
	Follow-up	54	43.52 (7.85)	43.42 [39.25, 48.75]	25.00, 60.00	[40.90, 45.32]			
UCLA Loneliness (ULS-8) (composite, 8–32)	Baseline	58	15.17 (5.17)	14.50 [10.46, 20.00]	8.00, 26.00	[13.92, 16.60]	0.49 (2, 53.0)	0.613	0.018 (0.14)
	Post	56	14.68 (4.87)	14.00 [10.75, 18.00]	8.00, 25.14	[13.59, 16.17]			
	Follow-up	54	14.86 (5.20)	14.00 [10.00, 19.75]	8.00, 26.00	[13.76, 16.54]			
Exercise Self-Efficacy (composite, 10–40)	Baseline	57	29.80 (6.92)	31.00 [27.00, 34.44]	10.00, 40.00	[27.89, 31.47]	1.39 (2, 54.9)	0.257	0.048 (0.23)
	Post	56	30.91 (4.35)	31.00 [28.00, 34.00]	22.00, 39.00	[29.70, 31.99]			
	Follow-up	55	30.34 (5.28)	31.00 [28.00, 34.00]	15.56, 40.00	[28.92, 31.69]			
Falls Efficacy (composite, 10–100)	Baseline	56	31.06 (22.55)	20.50 [13.00, 46.53]	10.00, 85.00	[26.35, 39.30]	1.79 (2, 51.0)	0.178	0.065 (0.27)
	Post	52	27.71 (18.31)	21.00 [14.75, 35.00]	10.00, 90.00	[24.28, 34.20]			
	Follow-up	51	28.48 (18.33)	20.00 [15.00, 39.50]	10.00, 72.00	[24.97, 35.69]			
Fear of Falling Severity (ordinal, 0–3)	Baseline	59	1.20 (1.06)	1.00 [0.00, 2.00]	0.00, 3.00	[0.93, 1.48]	0.39 (2, 53.7)	0.681	0.014 (0.12)
	Post	53	1.23 (0.99)	1.00 [0.00, 2.00]	0.00, 3.00	[0.93, 1.46]			
	Follow-up	54	1.26 (0.99)	1.00 [1.00, 2.00]	0.00, 3.00	[1.00, 1.52]			
Fear of Falling Activity Restriction (binary, 0/1)	Baseline	59	0.51 (0.50)	1.00 [0.00, 1.00]	0.00, 1.00	[0.37, 0.63]	2.05 (2, 52.6)	0.139	0.072 (0.28)
	Post	52	0.62 (0.49)	1.00 [0.00, 1.00]	0.00, 1.00	[0.46, 0.73]			
	Follow-up	55	0.62 (0.49)	1.00 [0.00, 1.00]	0.00, 1.00	[0.49, 0.75]			
Emotional Distress (PROMIS T-score)	Baseline	60	50.01 (8.44)	51.20 [46.20, 55.52]	37.10, 68.30	[47.83, 52.19]	0.01 (2, 55.2)	0.993	0.000 (0.02)
	Post	56	49.74 (8.80)	50.50 [43.30, 56.43]	37.10, 69.30	[47.74, 52.26]			
	Follow-up	55	50.01 (8.65)	52.30 [44.75, 55.75]	37.10, 67.40	[47.82, 52.31]			
Quality of Life (single item, 1–7)	Baseline	60	5.35 (1.34)	5.50 [4.75, 6.00]	1.00, 7.00	[5.00, 5.70]	0.45 (2, 56.7)	0.643	0.015 (0.12)
	Post	56	5.48 (1.32)	6.00 [5.00, 6.25]	2.00, 7.00	[5.12, 5.81]			
	Follow-up	55	5.42 (1.12)	6.00 [5.00, 6.00]	3.00, 7.00	[5.12, 5.71]			
Pain Interference (PROMIS T-score)	Baseline	58	58.91 (8.66)	59.90 [55.60, 65.20]	41.60, 75.60	[56.65, 61.13]	0.29 (2, 54.4)	0.748	0.011 (0.10)
	Post	53	58.07 (9.47)	58.50 [53.90, 65.20]	41.60, 75.60	[55.80, 60.81]			
	Follow-up	55	58.03 (9.38)	59.90 [52.95, 63.80]	41.60, 75.60	[55.70, 60.59]			

Note. LMMs estimated via REML with unstructured covariance and Satterthwaite df. PASIPD = Physical Activity Scale for Individuals with Physical Disabilities. Raw and model-estimated means for PASIPD differ due to log transformation. M = back-transformed estimated marginal mean (model-based); the median, IQR (Q1, Q3), and range are observed values back-transformed to MET h/wk. Because PASIPD was analyzed on the log+1 scale, the marginal mean is a geometric-type mean and may fall below the observed median for zero-inflated activity domains. 95% CIs are for the EMM (z = 1.96).

**Table 2:** Social Connectedness subscale scores across time: descriptive statistics and linear mixed model (LMM) results.

Subscale	Items	Baseline *M* (*SD*)	Post-Intervention *M* (*SD*)	Follow-Up *M* (*SD*)	LR χ^2^	Pairwise
Network Size	1–4	16.57 (3.36)	16.86 (3.23)	16.51 (3.21)	1.32	—
Social Support	5–7 ᴿ	7.17 (1.49)	7.29 (1.45)	7.18 (1.51)	0.59	—
Loneliness & Isolation	8–10	7.23 (1.81)	7.25 (1.73)	7.46 (1.73)	1.42	—
**Social Participation**	11–15 ᴿ	11.76 (3.54)	13.10 (3.92)	12.44 (3.46)	8.55 *	Pre vs. Post *

Note. N = 60. LMMs fit with REML estimation and random intercepts. LR χ^2^ = likelihood ratio test (df = 2). Pairwise comparisons Bonferroni-corrected. ᴿ = reverse-coded items. * *p* < 0.05.

## Data Availability

The original data presented in the study are openly available from the Inter-university Consortium for Political and Social Research at https://www.openicpsr.org/openicpsr/project/218343/version/V1/view (accessed on 11 March 2025) [80].

## Supplementary Materials

The following supporting information can be downloaded at: https://www.mdpi.com/article/10.3390/healthcare14121756/s1, TREND Statement Checklist [102].

## Abbreviations

The following abbreviations are used in this manuscript:

TCAFP	Tai Chi for Arthritis and Fall Prevention
LMM	Linear mixed models (LMMs)
PASIPD	Physical Activity Scale for Individuals with Physical Disabilities
CI	Confidence interval
PA	Physical activity
EMM	Estimated marginal means
REML	Restricted maximum likelihood
ESES	Exercise Self-Efficacy Scale
FES	Falls Efficacy Scale
